# Root engineering in maize by increasing cytokinin degradation causes enhanced root growth and leaf mineral enrichment

**DOI:** 10.1007/s11103-021-01173-5

**Published:** 2021-07-17

**Authors:** Eswarayya Ramireddy, Hilde Nelissen, Jan Erik Leuendorf, Mieke Van Lijsebettens, Dirk Inzé, Thomas Schmülling

**Affiliations:** 1 Institute of Biology/Applied Genetics, Dahlem Centre of Plant Sciences (DCPS), Freie Universität Berlin, Albrecht-Thaer-Weg 6, 14195 Berlin, Germany; 2 Biology Division, Indian Institute of Science Education and Research (IISER) Tirupati, Tirupati, 517507 Andhra Pradesh India; 3 Department of Plant Biotechnology and Bioinformatics, Ghent University, 9052 Ghent, Belgium; 4 VIB Center for Plant Systems Biology, 9052 Ghent, Belgium

**Keywords:** Cytokinin, Cytokinin oxidase/dehydrogenase, Maize, Mineral nutrition, Root system, *Zea mays*

## Abstract

**Key message:**

Root-specific expression of a cytokinin-degrading *CKX* gene in maize roots causes formation of a larger root system leading to higher element content in shoot organs.

**Abstract:**

The size and architecture of the root system is functionally relevant for the access to water and soil nutrients. A great number of mostly unknown genes are involved in regulating root architecture complicating targeted breeding of plants with a larger root system. Here, we have explored whether root-specific degradation of the hormone cytokinin, which is a negative regulator of root growth, can be used to genetically engineer maize (*Zea mays* L.) plants with a larger root system. Root-specific expression of a *CYTOKININ OXIDASE/DEHYDROGENASE* (*CKX*) gene of *Arabidopsis* caused the formation of up to 46% more root dry weight while shoot growth of these transgenic lines was similar as in non-transgenic control plants. The concentration of several elements, in particular of those with low soil mobility (K, P, Mo, Zn), was increased in leaves of transgenic lines. In kernels, the changes in concentration of most elements were less pronounced, but the concentrations of Cu, Mn and Zn were significantly increased in at least one of the three independent lines. Our data illustrate the potential of an increased root system as part of efforts towards achieving biofortification. Taken together, this work has shown that root-specific expression of a *CKX* gene can be used to engineer the root system of maize and alter shoot element composition.

**Supplementary Information:**

The online version contains supplementary material available at 10.1007/s11103-021-01173-5.

## Introduction

Roots fulfil important functions for plants, including anchoring in the soil and providing access to soil nutrients and water. Plant roots are known to be an important factor determining the agricultural performance of crop plants. However, because root traits are difficult to assess and select for, their potential for crop plant improvement has as yet not been fully exploited and numerous details of factors and genes controlling root system traits remain underexplored (Lynch and Brown 2012; White et al. 2013; Rogers and Benfey 2015; Hochholdinger 2016; Koevoets et al. 2016; Bray and Topp 2018).

Root traits are also of vital importance for maize (*Zea mays* L.), which is one of the most important cereal grains grown worldwide (Shiferaw et al. 2011). A recent study using a machine learning program for trait analysis of 57 commercial maize hybrids concluded that root traits were most important for predicting yield (Tucker et al. 2020). Several root traits have been identified in maize that are relevant for the exploration of soil resources, particularly in resource-poor environments (Lynch and Brown, 2012; York et al. 2013). For example, drought tolerance is associated with an increase in rooting depth and water acquisition from the subsoil (Gao and Lynch 2016). Plants with improved root traits may contribute to relieve a major constrain for the production of maize in developing countries, which is low soil fertility and high water requirement (Rusinamhodzi et al. 2011; Reynolds et al. 2015; ten Berge et al. 2019).

It is thought that selection for yield has indirectly selected also for root traits contributing to enhance maize yield (Hammer et al. 2009; Bray and Topp 2018). However, the genes regulating these traits are mostly unknown although a large number of QTLs associated with root traits have been identified in maize (Hund et al. 2011; Bray and Topp, 2018). However, so far only eight maize genes were identified that are involved in regulating root growth and development but their individual relevance for plant performance is as yet not clear (Hochholdinger et al. 2018). To understand and fully exploit the potential of roots for crop improvement, it would be important to compare near isogenic lines that differ principally in their root system while shoot growth and development should not be altered. To achieve this, we explore here an experimental approach based on changing the endogenous content of the plant hormone cytokinin by genetic engineering.

The hormone cytokinin is a well-known inhibitor of root elongation and branching (Werner et al. 2001, 2003; Chang et al. 2013, 2015). Cytokinin is degraded by cytokinin oxidases/dehydrogenases (CKX), which are encoded by small gene families in plants including maize (Schmülling et al. 2003). Enhanced degradation of cytokinin by enhanced expression of a *CKX* gene in roots caused the formation of a larger root system in *Arabidopsis thaliana* (Werner et al. 2010), barley (Ramireddy et al. 2018a,b), oilseed rape (Nehnevajova et al. 2019), rice (Gao et al. 2014) and chickpea (Khandal et al. 2020). Thus, it was shown repeatedly that a single dominant gene may be used to regulate a complex trait such as root system size. *CKX* transgenic plants with a larger root system were shown to respond less sensitive than the cognate wild-type plants to drought (Werner et al. 2010; Ramireddy et al. 2018a) underpinning the beneficial effect of a larger root system under water deficit (Comas et al. 2013; Gao and Lynch 2016; Klein et al. 2020). A surprising common feature of these plants had been the higher content of distinct micro- and macro-elements in their shoots. In particular, the concentration on zinc (Zn), a microelement missing in the diet of about 2 billion people, was found to be significantly increased in the seeds of *CKX* transgenic barley plants grown in the greenhouse and in the field (Ramireddy et al. 2018a,b). Consequently, it has been proposed that root enhancement might contribute to a sustainable solution for nutrient deficiencies (Werner et al. 2010; Ramireddy et al. 2018a; Gao et al. 2019; Khandal et al. 2020).

Here, we have explored the potential to engineer the maize root system by enhanced root-specific expression of a *CKX* gene. Transgenic maize lines formed a larger root system without reducing shoot growth demonstrating the potential of root engineering in maize. The shoots of these plants contained higher concentrations of several essential elements underpinning the role of cytokinin in regulating mineral nutrition.

## Results

### Generation of transgenic maize plants with enhanced *CKX* gene expression in roots

**Fig. 1:**
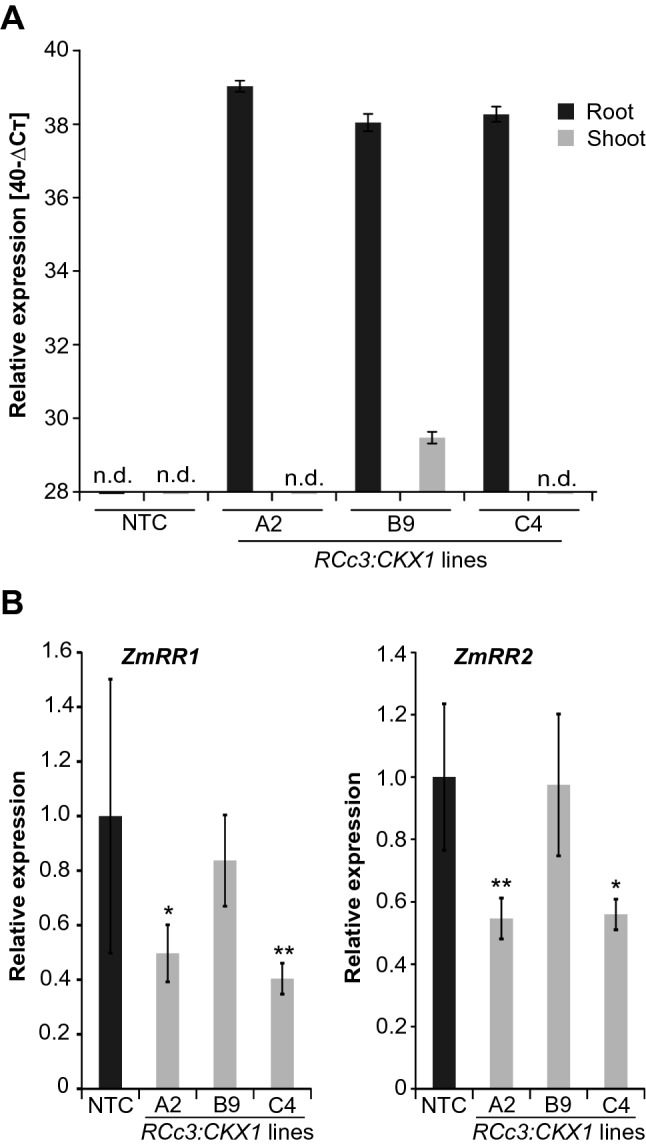
**A** Root-specific expression of *pRcc3:CKX1* in transgenic maize. The expression analysis of *CKX1* was carried out by qRT-PCR. Total RNA was extracted from roots and shoots (leaves) of 14-days-old plants. Relative expression of the transgene is shown as 40-ΔCT value, with 28 being the threshold value for expressed genes. A2, B9 and C4 are independent transgenic lines. **B** Expression of primary cytokinin response genes in roots. The expression of *ZmRR1* and *ZmRR2* was analysed in roots of 7-days-old transgenic and NTC seedlings. For A and B *ß-TUB* (*NP_001105457*) and *EF1a* (*NM_001112117*) were used as reference genes to normalize expression levels. Three (**A**) and four (**B**) biological replicates were analyzed for each genotype. Data are means ± SD. The significance of differences between the transgenic lines and NTC in (**B**) was calculated by One-way ANOVA (*ZmRR1*) or Kruskal–Wallis non-parametric test (*ZmRR2*) with Benjamini Hochberg correction (*, *p* < 0.05; **, *p* < 0.01). NTC, non-transgenic control

We chose the *RCc3* promoter of rice (Xu et al. 1995) to achieve root-specific expression of the *Arabidopsis CKX1* gene in the maize inbred line B104 (Coussens et al. 2012). The *RCc3* promoter has been used to drive root-specific expression of several genes in rice and barley (Jeong et al. 2010; Gao et al. 2014; Ramireddy et al. 2018a). The maize ortholog of rice *RCc3* (*GRMZM2G410338_T01*) was also shown to be expressed in a root-specific manner (Sekhon et al. 2011). The *CKX1* gene was chosen as its root-specific expression enhanced root growth in tobacco, *Arabidopsis* (Werner et al. 2010) and barley (Ramireddy et al. 2018a). Self-fertilization of transgenic T1 plants in which the T-DNA was in a single locus resulted in homozygote lines. Expression of the transgene was quantified by qRT-PCR using RNA isolated from entire shoots and roots. Three independent transgenic lines (A2, B9, C4) showing a high expression of *CKX1* in roots and no or a much lower expression in shoots (Fig. 1A) were selected for further analysis.

In order to explore the cytokinin status of the roots of these lines, the expression levels of two primary cytokinin response genes, *ZmRR1* (*Zea mays response regulator1*) and *ZmRR2* (Sakakibara et al. 1999; Asakura et al. 2003) were determined in roots. The results showed a significantly reduced expression of *ZmRR1* and *ZmRR2* in roots of two of the three independent transgenic lines as compared to the non-transgenic control (NTC) (Fig. 1B). The reduction of the expression levels of the two maize cytokinin response genes is comparable to the decrease observed in cytokinin signaling mutants of *Arabidopsis* and rice (Argyros et al. 2008; Worthen et al. 2019). These results therefore suggest that enhanced expression of Arabidopsis *CKX1* causes a reduced cytokinin status in maize roots.

### Transgenic maize plants exhibit enlarged root systems

In order to analyse the consequences of enhanced expression of the *CKX1* gene in roots of maize we compared transgenic lines with a NTC at different developmental stages and upon growth in different conditions, i.e. plants were grown either in a hydroponic system or in soil.

**Fig. 2:**
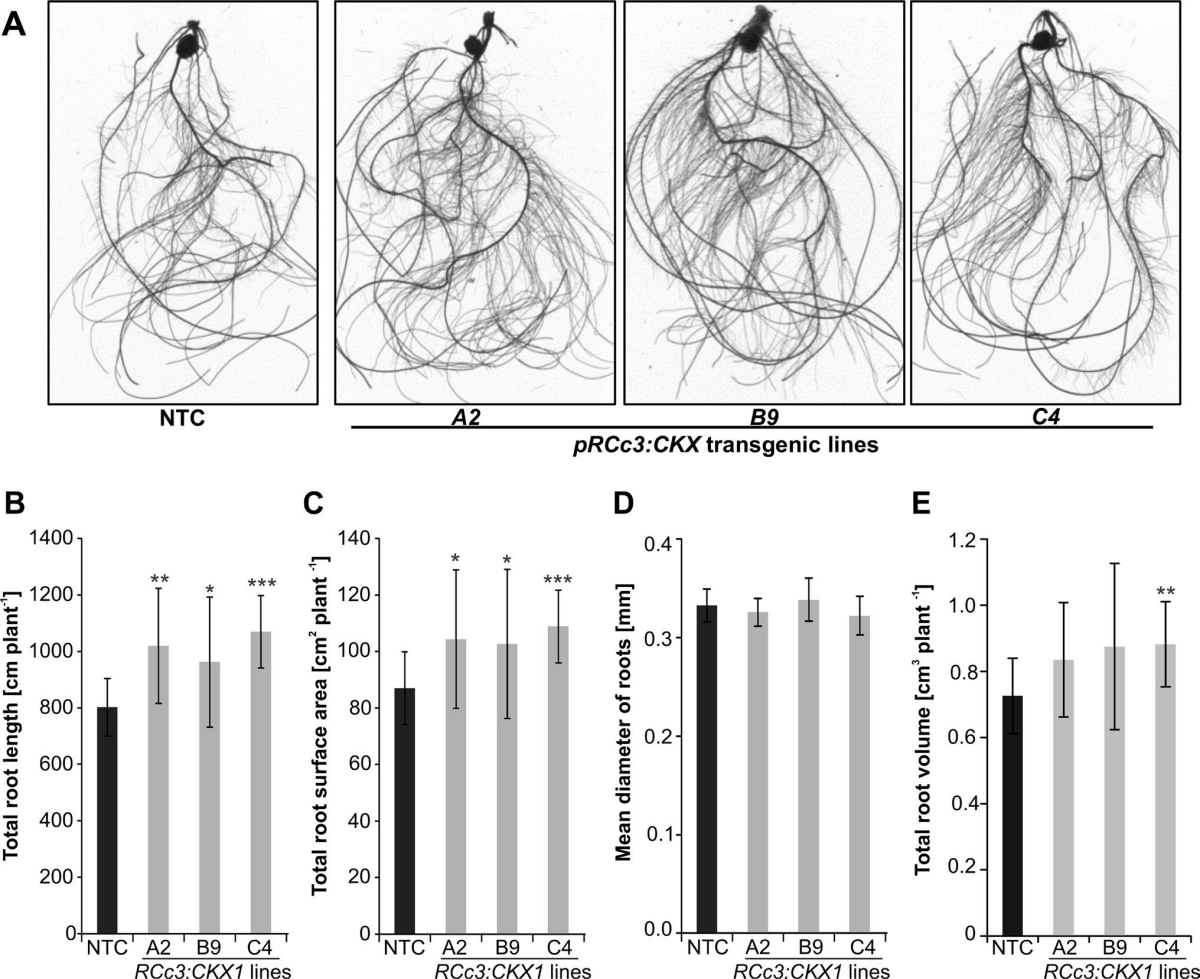
Root–specific expression of *pRCc3:CKX1* increases the root system size of maize. **A** Root phenotype of 10-days-old lines grown in hydroponic culture. Representative images of root systems from individual plants are shown. **B** Total root length; **C** total root surface area; **D** mean diameter of roots, and **E** root volume of 10-days-old plants. Total root length, surface area, mean diameter and root volume were calculated using the WinRHIZOTM software. Data in (**B**–**E**) represent means ± SD (*n* = 12). Asterisks indicate statistically significant differences compared to NTC as determined by two-tailed Student's *t*-test (*, *p* < 0.05; **, *p* < 0.01; ***, *p* < 0.001). NTC, non-transgenic control

Ten days old seedlings of all three independent transgenic lines grown in a hydroponic system showed a larger root system as compared to NTC (Fig. 2). Total root length was increased between 20 and 33% (Fig. 2B) and total root surface area was increased between 18 and 23% in the three transgenic lines (Fig. 2C), while the root diameter was not altered (Fig. 2D). Consequently, the mean root volume of transgenic plants was 15–21% larger than that of the NTC (Fig. 2E).

**Fig. 3:**
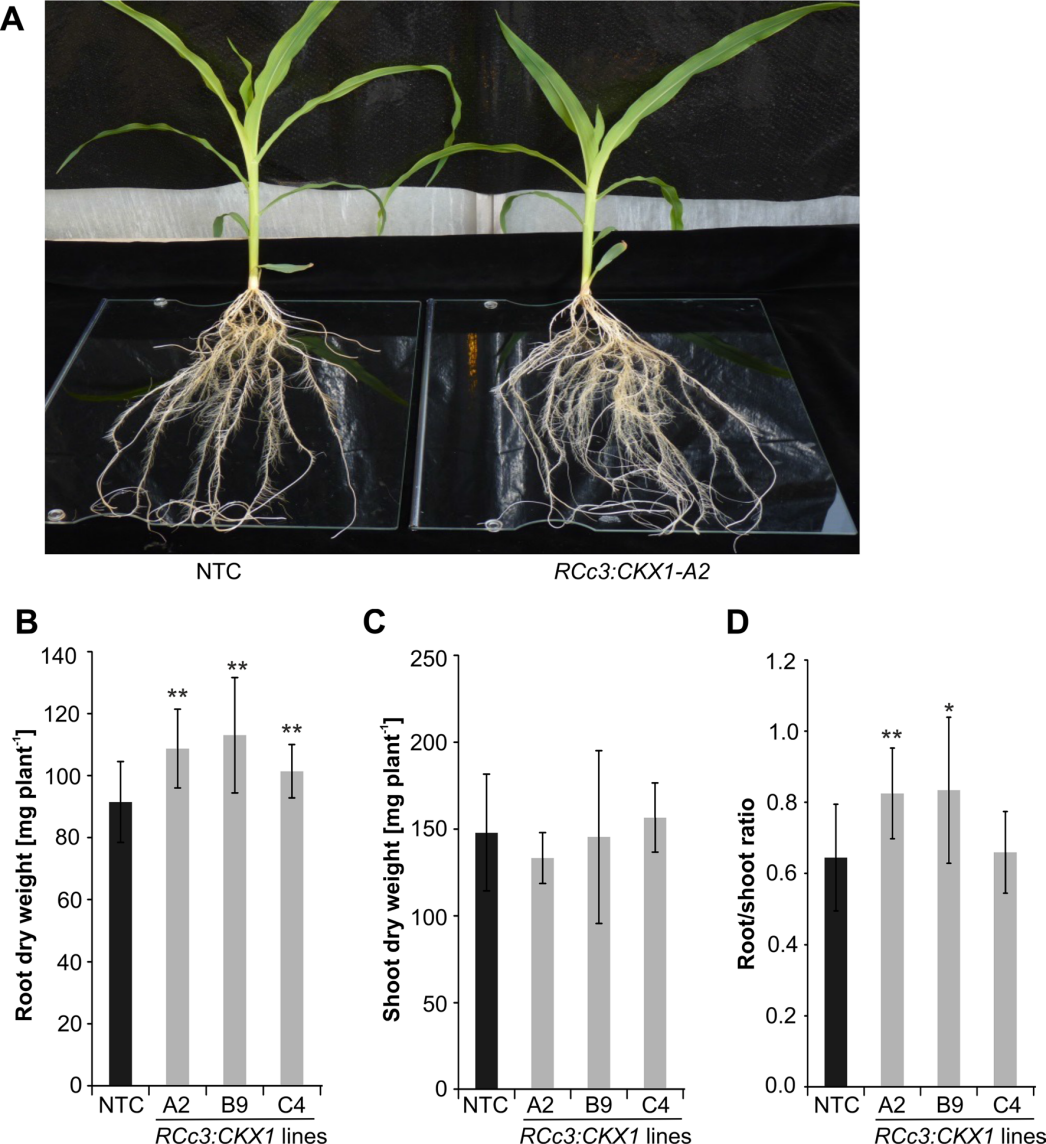
Root-specific expression of *pRCc3:CKX1* increases maize root system size in hydroponic culture. **A** Root phenotype of 18-d-old plants grown in hydroponic culture. **B** Root dry weight. **C** Shoot dry weight. **D** Root/shoot ratio. Data in (**B**–**D**) represent means ± SD (*n* = 12). Asterisks indicate statistically significant differences compared to NTC as determined by two-tailed Student's *t-*test (*, *p* < 0.05; **, *p* < 0.01). NTC, non-transgenic control

Analysis of 18-d-old plants grown in a hydroponic system (Fig. 3A) confirmed the increased root growth in transgenic lines. At this stage, root dry weight of the transgenic lines was increased by 11–23% (Fig. 3B). In contrast, shoot dry weight of transgenic lines did not differ significantly from the control line (Fig. 3C). This differential growth resulted in an increased root-to-shoot ratio in two of the three transgenic lines (Fig. 3D).

**Fig. 4:**
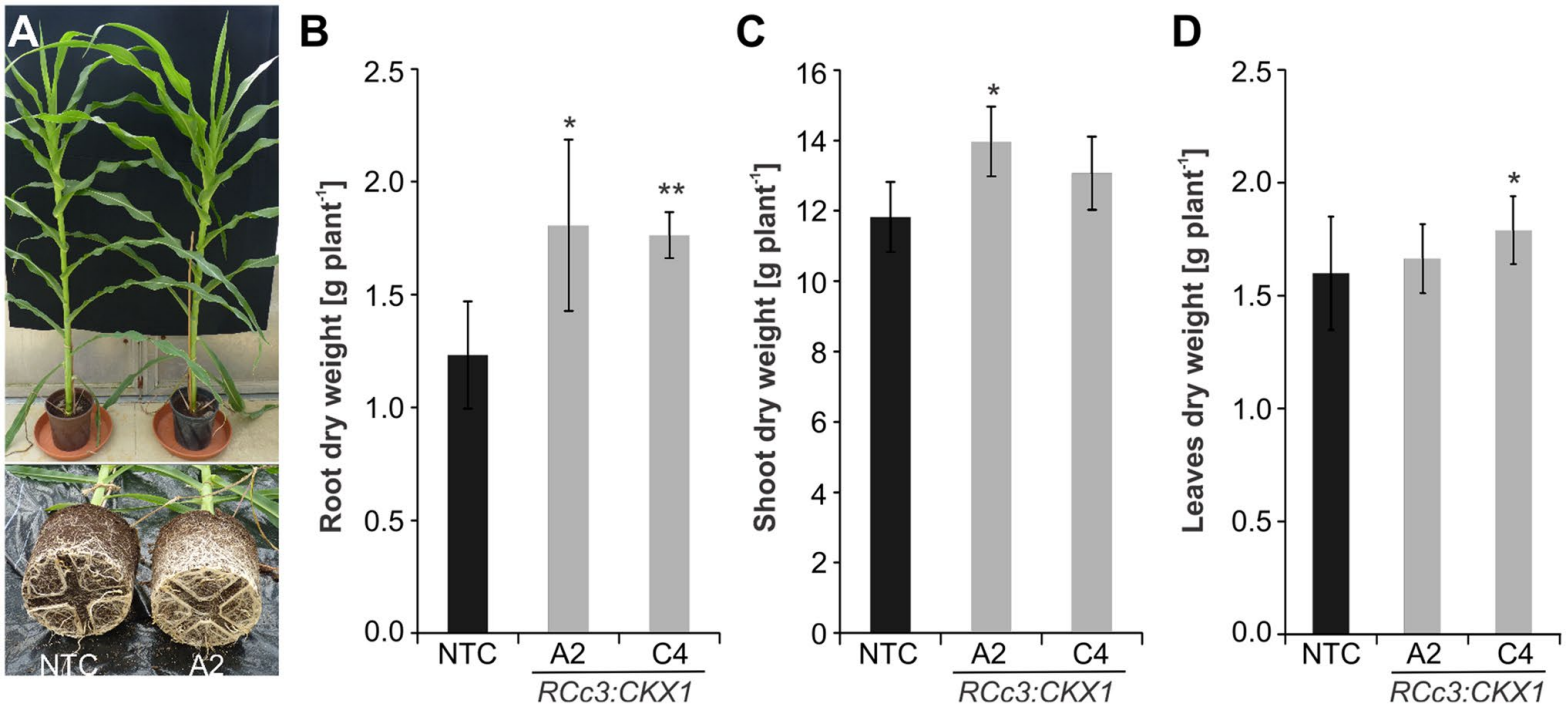
*pRCc3:CKX* transgenic plants have normal shoot growth. **A** Shoots and roots of five-week-old plants grown in soil-filled pots. A representative image from shoot and root systems of individual plants is shown. **B** Root biomass. **C** Shoot biomass. **D** Leaf biomass of four-week-old plants. Data are means ± SD (*n* = 8–10). Asterisks indicate statistically significant differences to the NTC as determined by two-tailed Student's *t*-test (*p < 0.05; **p < 0.01). NTC, on-transgenic control

Finally, root and shoot dry weight of five-week-old soil-grown plants were compared. Visual inspection of these plants revealed no changes of shoot size while the root system of transgenic lines showed enhanced growth (Fig. 4A). The root dry weight of the two transgenic lines that were tested was 1.80 ± 0.30 g (line A2) and 1.76 ± 0.10 g (line C4) while roots of the control line had on the average a dry weight of 1.23 ± 0.20 g (Fig. 4B), representing an increase of 43–46% in the transgenic lines. The shoot and leaf dry weight was similar to the control or increased by 12% (Fig. 4C, D). Several leaf growth parameters were analysed in more detail (Supplemental Fig. S1). The results indicated that there was no difference of the leaf elongation rate between transgenic and non-transgenic maize plants (Supplemental Fig. S1A, S1B). However, several parameters including leaf length (+ 4%), leaf blade area (+ 11%) and fresh weight (+ 12%) were slightly increased in both or at least one of the transgenic lines when compared to their respective NTCs (Supplemental Fig. S1C to S1F). Collectively, this analysis has shown that increased *CKX* gene expression in the roots of maize causes increased root growth with similar or slightly increased shoot growth.

### Root enhancement caused an increased accumulation of distinct elements in the shoot

One important function of roots is the uptake of elements from the soil. In order to explore the eventual impact of an increased root system size on the element concentration in the shoot, we measured the concentrations of 17 different elements in leaves of four-week-old soil-grown plants as well as in seeds.

**Fig. 5:**
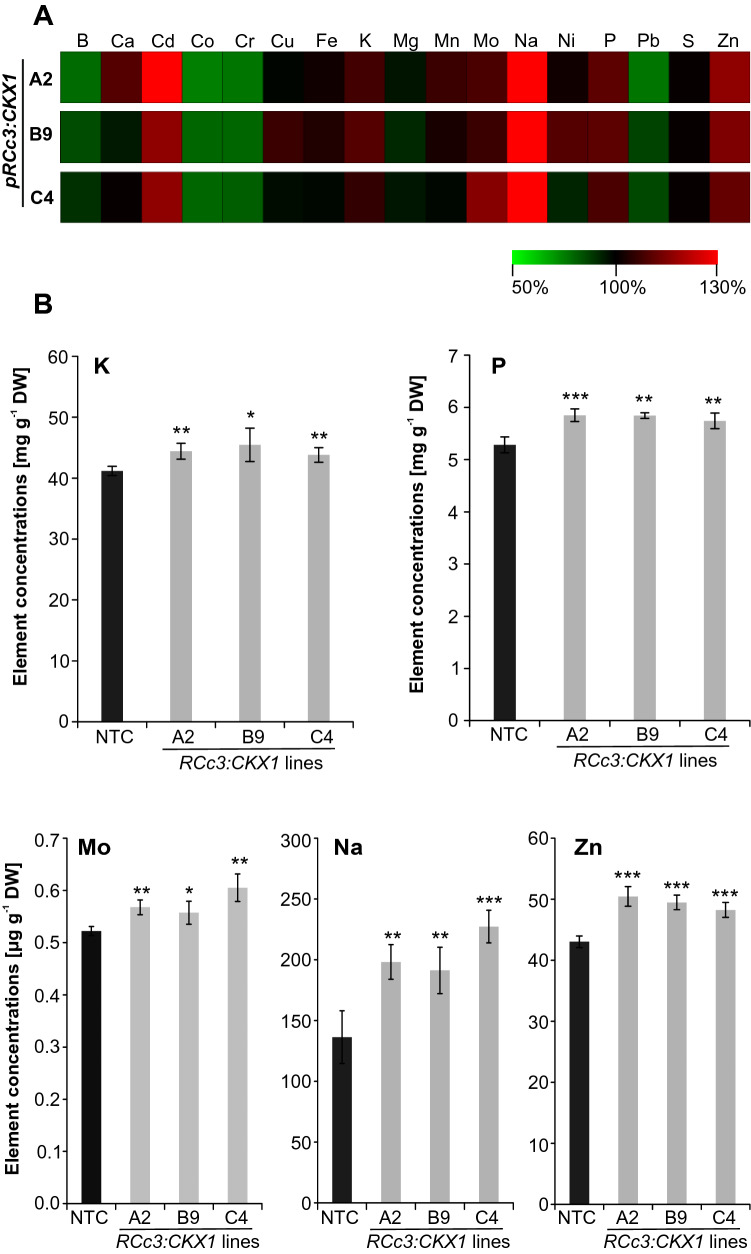
Mineral element concentrations in leaves of *pRCc3:CKX1* transgenic maize plants. **A** Relative changes in mineral element concentrations in transgenic lines compared to non-transgenic control plants (NTC). The concentration of each mineral element in NTC leaves was set to 100% and relative differences in transgenic lines are shown in a heat map generated using Multi-experiment Viewer v4.9 (Saeed et al. 2003). The complete data set is shown in Supplemental Table S1. **B** Concentrations of different mineral elements in leaves of four-weeks-old soil-grown plants. Four biological replicates for each genotype were analysed, each containing shoots from 2–3 plants. Data shown are means ± SD. Asterisks indicate significant differences to the NTC as determined by two-tailed Student's *t*-test (*, *p* < 0.05; **, *p* < 0.01; ***, *p* < 0.001). NTC, non-transgenic control

As shown in Fig. 5A, in the leaves of the *CKX* overexpressing maize several elements show consistent changes as compared to the leaves of NTC plants. The element showing the strongest increase in concentration in all three transgenic lines was sodium (Na), which increased by 40–67% in the leaves of CKX maize plants compared to the control (Fig. 5 and Supplemental Table S1). Among the other macro-elements, the concentrations of potassium (K) and phosphorus (P) were consistently and significantly increased by ~ 10% in the leaves of transgenic plants (Fig. 5B, Supplemental Table S1). In contrast, the content of other macro-elements like S and Mg was either similar or slightly reduced compared to control plants (Fig. 5A, Supplemental Table S1).

In the case of micro-elements, the molybdenum (Mo) and zinc (Zn) concentrations were increased by 7–16% and 12–17% (Fig. 5B), respectively, whereas the concentration of boron (B) was decreased compared to the control plants (Supplemental Table S1).

The concentrations of heavy metals were changed as well in leaves of transgenic plants. The concentration of cobalt (Co), chromium (Cr) and lead (Pb) were consistently decreased by 20–40% in the transgenic plants whereas cadmium (Cd) increased by 17–30% compared to NTC plants.

**Fig. 6:**
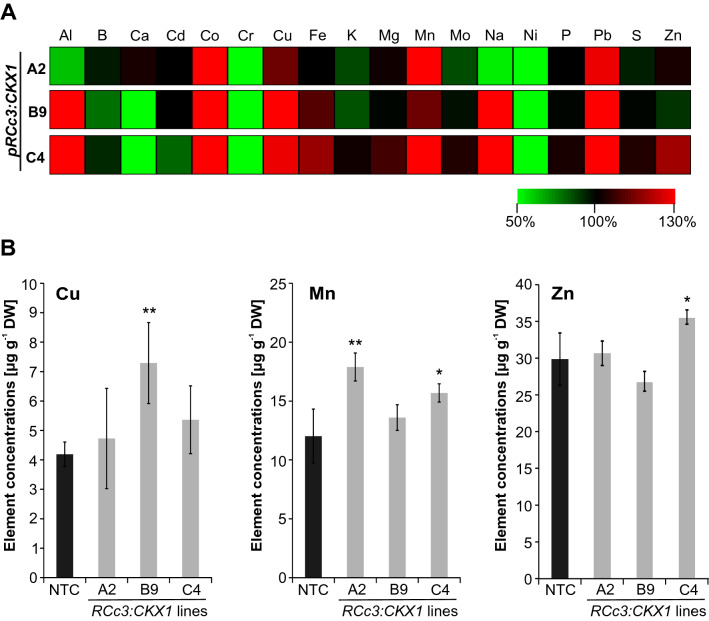
Mineral element concentrations in seeds of *pRCc3:CKX1* transgenic maize plants. **A** Relative changes in mineral element concentrations in transgenic lines compared to non-transgenic control (NTC) plants. The concentration of each mineral element in NTC seeds was set to 100% and relative differences in transgenic lines are shown in a heat map generated using Multi-experiment Viewer v4.9 (Saeed et al. 2003). The complete data set is shown in Supplemental Table S2. **B** Concentrations of copper (Cu) and manganese (Mn) and zinc (Zn) in seeds of transgenic lines in comparison to NTC seeds. Four biological replicates for each genotype were analysed, each containing seeds from 2–3 plants. Data shown are means ± SD. Asterisks indicate significant differences to the NTC as determined by two-tailed Student's *t-*test (*, *p* < 0.05; **, *p* < 0.01). NTC, non-transgenic control

Next, we analyzed whether the changed element concentrations in leaves would be reflected by similar changes in maize kernels. Figure 6A shows that the changes of element concentration in kernels clearly differed from those measured in leaves. Seeds from transgenic lines contained 13–70% higher concentrations of copper (Cu), which was also found in barley (Ramireddy et al. 2018a) and 13–49% higher concentrations of manganese (Mn). The concentration of zinc was significantly increased by 19% in one of the transgenic lines while the other two lines showed a similar or slightly decreased zinc concentration compared to the control (Fig. 6 and Supplemental Table S2). When it comes to heavy metals, the concentration of Cr and Ni was decreased by ~ 70–80%, and the concentration of Co and Pb increased by ~ 40–70% in seeds of CKX maize compared to control plants, but all concentrations were very low (Fig. 6 and Supplemental Table S2).

## Discussion

Maize plants overexpressing a cytokinin-degrading *CKX* gene in their roots formed a larger root system with longer roots, an increased surface area and enhanced dry weight. Importantly, root enhancement as documented for different developmental stages was significant for all independent transgenic lines in all stages (Figs. 2B, C; 3B; 4B). This showed that the regulatory role of cytokinin in roots is similar in maize as in other species and has been conserved during evolution and domestication.

The increased root system was already established at the seedling stage showing that cytokinin regulates the development of embryonic roots. The size of the early root system of maize seedlings is associated with nutrient acquisition and drought resistance (Kumar et al. 2012; Abdel-Ghani et al. 2013; Li et al. 2015). Early root growth is also a relevant trait supporting seedling establishment and vigor (Sanguinetti et al. 1998). The root enhancement in *CKX* overexpressing maize was persistent until later developmental stages as well as under different growth conditions indicating that development of post-embryonic roots establishing the main part of the root system of adult maize plants is also under control of cytokinin. The increase of the different root traits (length, surface area, number of tips, root volume, dry weight) was in the range of 23 to 46%, which is in a similar range as the increase found in *CKX* overexpressing rice and *CKX* overexpressing barley (Gao et al. 2014; Ramireddy et al. 2018a). For comparison, the minimal and maximal root dry weights of commercial maize hybrids showed about 20% difference from the average value of all 57 hybrids (Tucker et al. 2020), indicating that the increase in root size of CKX maize covers at least a substantial part of the variation and may even exceed it. In a comparison of drought-sensitive and drought-tolerant maize varieties, an increase of root length (33%) was shown to be associated with increased drought tolerance (Rosa et al. 2019). This suggests that the size increase of the CKX maize root system is functionally relevant and might be advantageous under drought. An even higher increase of root system size might be achieved by overexpressing a CKX protein that is targeted to the extracellular space and not to the endoplasmatic reticulum as CKX1 (Niemann et al. 2018) and thus has access to a different cytokinin pool (Werner et al. 2010; Ramireddy et al. 2018a, b).

Importantly, root enhancement did not negatively impact shoot growth and development of CKX maize. Instead, there was rather a tendency to improved shoot growth in these plants (Fig. 4; Supp. Fig S1), indicating that plants do not suffer from source limitation but sufficient carbon is fixed to support growth of a larger root system. This is an important result as source limitation and the consequential negative impact of root enhancement on shoot growth could restrict the engineering of plants with a larger root system. Notably, it was shown in Arabidopsis that combining root and shoot engineering may even lead to plants with a larger root system and larger shoots (Vercruyssen et al. 2011).

The concentration of a number of several elements (K, P, Mo, Na and Zn) was significantly increased in leaves of all three independent CKX maize lines (Fig. 5). The macroelements showing consistently an increased concentration in leaves of all three CKX lines were phosphorus (+ 9–11%) and potassium (+ 6–8%). This confirms that root growth of maize is particularly crucial for the uptake of immobile nutrients such as phosphorus and potassium (Lynch 2011; Rosa et al. 2019). A role for cytokinin in regulating the response to the availability of phosphorus and potassium has been shown in *Arabidopsis* (Franco-Zorrilla et al. 2005; Nam et al. 2012).

A third macroelement that was increased between 40 and 67% in the leaves of CKX maize plants was sodium. Sodium is most often seen as problematic because of increasing soil salinity but it is known to be essential for plants that perform C_4_ or CAM photosynthesis and it may replace K to some extent (Adams and Shin 2014). In sorghum, a close relative of maize, an increased concentration of Na^+^ ions in leaves was shown to activate the expression of phosphoenolpyruvate carboxylase‐kinase (PEPCase‐K) under light and dark conditions. PEPCase-K is crucial for the carbon fixation efficiency of the C_4_ photosynthesis pathway (García-Mauriño et al. 2003). In how far the strongly increased sodium content of leaves in CKX maize affects physiology remains to be determined.

The concentrations of only two microelements were consistently changed in leaves, molybdenum (+ 7–16%) and zinc (+ 12–17%). Molybdenum is required among others for redox enzymes such as nitrate reductase and molybdenum deficiency is common in many different types of soil (Kaiser et al. 2005), often resulting in nitrate accumulation (Kovács et al. 2015).

The increase in shoot zinc concentration in CKX maize is of special interest as around two billion people worldwide suffer from nutritional deficiency of zinc and methods to achieve biofortification are needed. Often the mainly plant-based diets are not a sufficient source of this essential element (Prasad 2013; Menguer et al. 2018). Maize is a staple crop in many countries and zinc deficiency in the kernels is the cause of zinc malnutrition in maize consumers. In most maize lines, there is a solid gap between zinc concentrations and biofortification target values (Zhao et al. 2020) making maize a suitable target for zinc biofortification. In CKX maize, the zinc increase in leaves occurred in all lines but only one out of three lines showed a 19% increase of zinc concentration in the seeds. However, the zinc concentration in this line was as high as 35.6 μg g^−1^ DW, which reaches almost the target concentration of 38 μg g^−1^ DW set by the HarvestPlus biofortification program (Bouis and Welch 2010). The International Maize and Wheat Improvement Center (CIMMYT) has identified maize varieties with more than 33 μg zinc g^−1^ DW and recommended these varieties for future zinc biofortification breeding programs (Maqbool and Beshir 2019). The increase in zinc concentration in CKX maize shows that root enhancement in maize may be part of a zinc biofortification strategy but that selection of suitable lines would be required.

Noteworthy, the increase of the zinc concentration in shoots was the only change that was consistently found in all species with an enhanced root system analysed so far, namely Arabidopsis, tobacco (Werner et al. 2010), oilseed rape (Nehnevajova et al. 2019), barley (Ramireddy et al. 2018a,b), rice (Gao et al. 2019) and chickpea (Khandal et al. 2020). The increases varied between 32 and 68% but were stable under different growth conditions, in different soils as well as in hydroponics. In barley it was found in plants grown in the green house as well as in plants grown in the field. This stability and the fact that the increase occurred in monocot and dicot crop plants shows that this is a very stable trait positively correlated with an increase in root biomass. In *Arabidopsis* and rice *CKX* plants expression of zinc transporter genes was upregulated suggesting that derepression of these genes is responsible for the increased Zn uptake (Werner et al. 2010; Gao et al. 2019).

In addition to zinc, there was an increase in copper (13–74%) and manganese (13–49%) in the kernels of some maize lines. Both are also essential trace elements required for human nutrition (White and Broadley 2009; Bouis and Welch 2010) but they are generally in sufficient quantity in human diets. Manganese is mainly known for its essential role for photosynthetic activity and as an activator and co-factor for several metallo-enzymes (Schmidt and Husted 2019). The seed manganese concentration has been shown to be important to support seedling growth and vigor in wheat, particularly in dry, calcareous, and sandy soils where manganese deficiency is commonly observed (Singh and Bharti 1985; Moussavi-Nik et al. 1997). Wheat plants grown from grains with an increased manganese concentration withstood the take‐all (white heads) disease better and yield was higher than in plants from the grains of the same cultivar with a lower manganese concentration (McCay‐Buis et al. 1995). Whether or not the manganese content of maize seeds has a similar impact on plant performance is not known.

The accumulation of increased concentrations of elements in shoot organs is a common feature of different model and crop plants with enhanced root systems due to a lowered cytokinin content (Werner et al. 2010; Ramireddy et al. 2018a; Nehnevajova et al. 2019; Gao et al. 2019; Khandal et al. 2020). However, the element accumulation profile of CKX maize showed some peculiarities such as an increase in potassium not found in most other species and a lack of increase in sulfur and calcium, which was detected in most other CKX plants. This distinct profile argues against a general and common reason for the increased shoot element contents of CKX plants, such as the larger soil volume that is explored or a regulation of transfer cell density by cytokinin (Andersen et al. 2018). The differential impact of root enhancement on uptake of minerals in different species seems at least partially be unlinked to the growth regulatory effect of cytokinin. It might rather be due to differential regulation of respective transporter genes by cytokinin as was noted above for zinc transporter genes (Werner et al. 2010; Gao et al. 2019).

Taken together, we have expanded the use of root-specific cytokinin degradation as a means to achieve root enhancement to the important crop plant maize. Despite the complexity of the maize root system, it was possible to expand its size by expressing a single dominant gene. This approach might partially overcome limitations caused by the numerous genes contributing to shape the maize root system (Bray and Topp 2018). The recent description of a set of root-specific promoters of maize with distinct spatio-temporal expression profiles (Li et al. 2019) will be helpful to refine and optimize the approach in order to design different root ideotypes of maize for crop improvement.

Evidently, further analysis of the molecular basis of the enhanced shoot element content and testing of CKX maize plants under field conditions is required. It will be particularly interesting to explore how CKX maize performs on soils with micronutrient deficiencies as they are common in arable soils of sub-Saharan Africa where simultaneous deficiencies of several microelements enhanced in CKX maize such as zinc, molybdenum and copper occur (Kihara et al. 2020).

## Experimental procedures

### Plant material and growth conditions

Immature embryos of the maize inbred line B104 were transformed by *Agrobacterium tumefaciens* co-cultivation (Coussens et al. 2012). In short, immature B104 embryos were co-cultivated with *A. tumefaciens* for three days followed by one-week growth on non-selective medium. Transformed embryogenic calli were subsequently selected on increasing concentrations of phosphinotricin. After shoot induction from the selected calli, transgenic T0 plants were transferred to soil. T0 plants were backcrossed once with B104 wild type, resulting in a collection of T1 seeds from independent transgenic events that were self-pollinated. Homozygote transgenic lines and non-transgenic siblings were identified by quantitative PCR performed on genomic DNA by iDNA Genetics (Norwich, UK). In all experiments except the evaluation of leaf growth, an outsegregated non-transgenic sibling of line C4 was used as a non-transgenic control (NTC). In the comparison of leaf growth (Fig. S1), we compared lines A2 and C4 with their respective outsegregated NTCs.

For phenotypic analysis maize plants were grown under controlled greenhouse conditions (26/20 °C; 16/8 h light/dark cycle; 500 μmol m^−2^ s^−1^ by metal halide lamps (HQI) supplemented with tungsten bulbs). Plants for leaf growth monitoring were grown under growth chamber conditions with controlled relative humidity (55%), temperature (24 °C day/18 °C night), and a light intensity of 170–200 μmol m^−2^ s^−1^ photosynthetic active radiation at the plant level in a 16/8 h (day/night) cycle.

### RNA isolation and quantitative real-time PCR analysis

Total RNA was extracted from tissues using TRIzol reagent (Invitrogen) following the manufacturer’s protocol. RNA was purified using the RNayes MinElute clean up kit (Qiagen). Removal of genomic DNA was achieved using RQ1 RNase-Free DNase (Promega). 2 µg of total RNA were taken for cDNA synthesis using the RevertAid First Strand cDNA Synthesis Kit of Fermentas (St. Leon-Rot, Germany) and oligodT-primers. To test cDNA yield, qPCR was performed using primers of the maize elongation factor 1α (*EF1α*; NM_001112117) and tubulin β-chain (*β-TUB*; NP_001105457) as maize reference genes (Lin et al. 2014). Supplemental Table S3 lists the primer sequences used in this study. The cDNA samples were used to determine *CKX1* transgene expression and cytokinin primary response genes *ZmRR1* (gene ID Zm00001d001865) and *ZmRR2* (gene ID Zm00001d026594) levels by quantitative real-time PCR according to Cortleven et al. (2014).

### Quantification of root system size and biomass

Maize seeds were germinated on soil and three days after germination seedlings were carefully lifted from the soil and cautiously washed to remove bound soil particles. Seedlings of similar size were transferred to a hydroponic system and cultivated for another seven days for root system size analysis, and 15 d for biomass quantification. For the hydroponic system 0.1 × Hoagland solution (1 mM KH_2_PO_4_, 0.5 mM KNO_3_, 0.4 mM Ca(NO_3_), 0.2 mM MgSO_4_, 0.1 mM FeNaEDTA, 0.01 mM H_3_BO_3_, 2 μM MnSO_4_, 0.2 μM ZnSO_4_, 0.2 μM CuSO_4_, 0.1 μM Na_2_MoO_4_ and 0.02 mM NaCI) was used (Krämer et al. 1996). 12 L nutrient solution per box was properly aerated and changed every second day. After harvest, roots and shoots were separated and their fresh weights were determined. Thereafter, samples were dried in an oven at 80 °C for 68 h and the dry weight was recorded. For root system size analysis by WinRHIZO™, roots were carefully lifted from the box and spread out in a root-positioning tray (20 × 30 cm) to minimize root overlap and scanned with a flatbed scanner (EPSON, EU-88, Japan). Greyscale images obtained in tiff format were analysed with WinRHIZO™ (Pro Version 2005a; Regent Instruments Inc., Canada). For quantification of root system size of soil-grown transgenic plants, soil-filled 30 cm diameter pots were used. After five weeks of growth in soil, plants were harvested and separated into shoots and roots. Roots were carefully washed to remove bound soil particles and aggregates. Samples were dried in an oven at 80 °C for 68 h and the dry weight was recorded.

### Quantification of leaf and seed element content

Quantification and analysis of leaf and seed elements was performed as described in Ramireddy et al (2018a). Briefly, seeds of three independent transgenic lines and the NTC were germinated on filter paper in vitro. Three-day-old seedlings were transferred to the greenhouse into an unfertilized (type 0) soil supplied by the company Einheitserde (Sinntal-Altengronau, Germany). Composition of unfertilized soil was tested and certified by Institut Koldingen GmbH (Sarstedt, Germany) as described by Drechsler et al. (2015). Plants were grown further for four weeks by supplementing equal amounts of fertilizer solution every second or third day depending on soil moisture. The fertilizer solution was based on the composition of modified Hoagland solution (2 M KNO_3_, 1 M NH_4_NO_3_, 1 M KH_2_PO_4,_ 2 M Ca(NO_3_)_2_ 4 H_2_O,_,_2 M MgSO_4_ 7 H_2_O, 100 μM Na-Fe-EDTA, 50 μM H_3_BO_3_, 50 μM MnSO_4_, 18.5 μM ZnSO_4_, 50 nM CuSO_4_, 50 nM CoCl_2_, 0.5 μM NaMoO_4_ and 2 mM MES). The solution was adjusted to pH 5.7 with 1 M KOH. Total leaves from four-week-old plants were dried for 72 h at 80 °C and grounded carefully. Then equal amounts of powder (1 g) were weighed into polytetrafluoroethylene tubes and digested with a HNO_3_ + H_2_O_2_ mixture in a pressurized microwave digestion system (MARS from CEM GmbH; Kamp-Lintfort, Germany). The concentrations of macro- and microelements were analyzed by inductively-coupled plasma optical emission spectrometry (ICP-OES, iCAP 6500 dual OES spectrometer; Thermo Fischer Scientific) with certified standard reference samples as control. The element content from seed samples was determined in a similar way as outlined above.

## Supplementary Information

Below is the link to the electronic supplementary material.

Supplementary file1 (PDF 441 kb)

Supplementary file2 (PDF 237 kb)
