# Supplementary material for: Root engineering in maize by increasing cytokinin degradation causes enhanced root growth and leaf mineral enrichment
Source: Plant Mol Biol. 2021 Jul 17;106(6):555–67. doi: 10.1007/s11103-021-01173-5 (PMC8338857; doi:10.1007/s11103-021-01173-5)
Supplement: Supplementary file 1 — Supplementary file1 (PDF 441 kb) [file 11103_2021_1173_MOESM1_ESM.pdf]

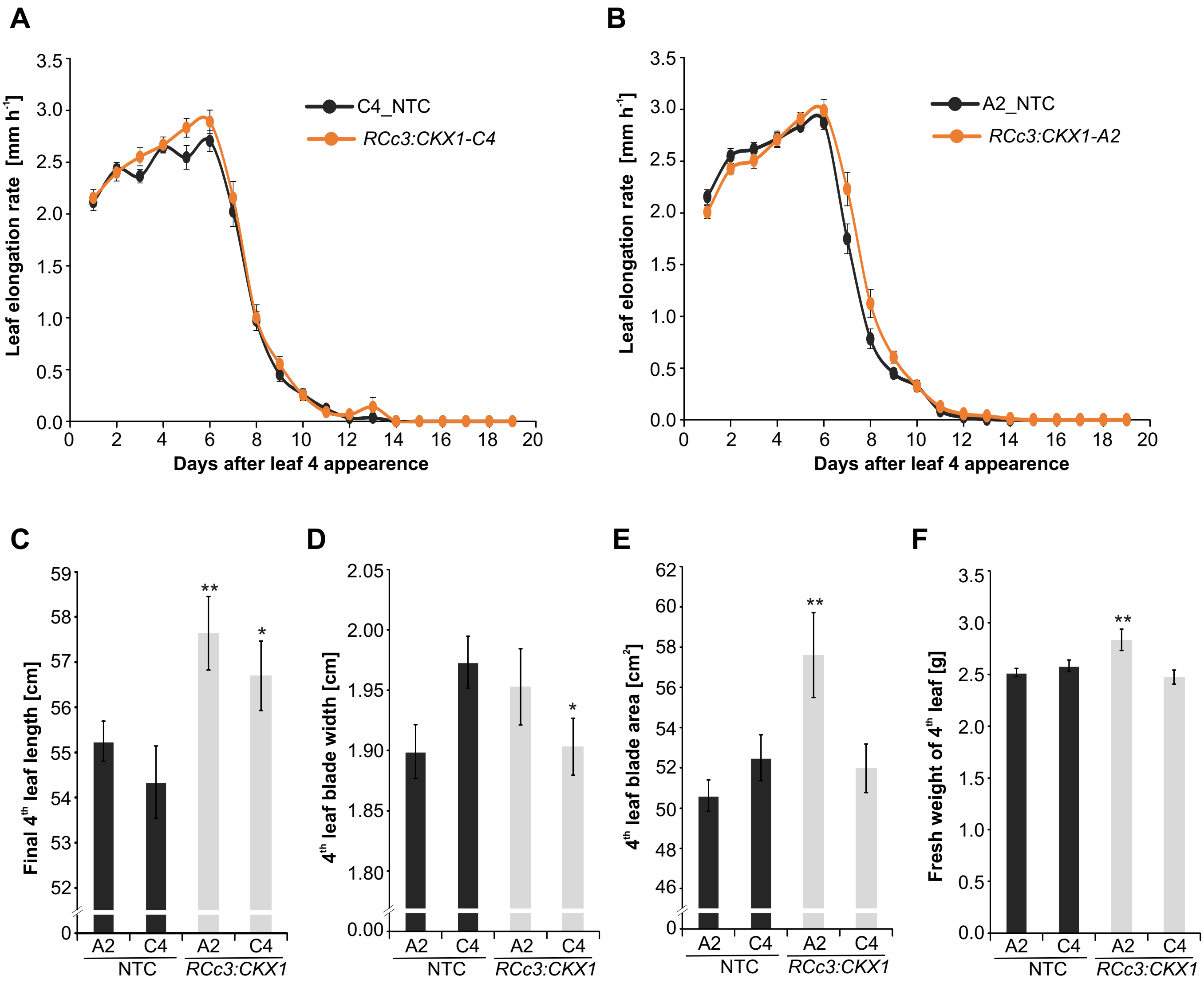

Supplemental Fig. 1. Analysis of maize leaf growth. A and B, Leaf elongation rate (LER) of leaf four from day one until day 19 of transgenic maize plants along with their respective non-transgenic control. C to F, Leaf four phenotypes. C, Leaf length; D, leaf width; E, leaf area and F, leaf biomass of transgenic plants compared to their respective non-transgenic control. 18 replicates for each genotype were analysed. Data represent means ± SD. Asterisks indicate statistically significant differences compared to their respective non-transgenic control as determined by two-tailed Student's *t*-test (*, $p < 0.05$; **, $p < 0.01$).
