# Supplementary material for: Root engineering in maize by increasing cytokinin degradation causes enhanced root growth and leaf mineral enrichment
Source: Plant Mol Biol. 2021 Jul 17;106(6):555–67. doi: 10.1007/s11103-021-01173-5 (PMC8338857; doi:10.1007/s11103-021-01173-5)
Supplement: Supplementary file 2 — Supplementary file2 (PDF 237 kb) [file 11103_2021_1173_MOESM2_ESM.pdf]

Supplemental Table S1. Element concentration in leaves of transgenic maize plants. Quantification of mineral elements was done in four-weeks-old soil-grown plants. Four biological replicates for each genotype were analyzed. Each biological replicate contained shoots from 2-3 plants. The statistical significance of differences from wild type was calculated using two-tailed Student's *t*-test (*, $p < 0.05$; **, $p < 0.01$; ***, $p < 0.001$). NTC, non-transgenic control; DW, dry weight. Part of the data are shown in Figure 5.

	**NTC**	***pRCC3:CKX1-A2***			***pRCC3:CKX1-B9***			***pRCC3:CKX1-C4***		
**Element**	Mean ±SE	Mean ±SE	% NTC	*t*-test	Mean ±SE	% NTC	*t*-test	Mean ±SE	% NTC	*t*-test
**B** (μg g^{-1} DW)	16.1 ±0.4	12.8 ±0.3	79	**	13.7 ±0.8	85		13.12 ±0.7	81	
**Ca** (mg g^{-1} DW)	11 ±0.2	12.2 ±0.7	110		10.4 ±0.4	95		11.0 ±0.4	101	
**Cd** (mg g^{-1} DW)	0.17 ±0.007	0.23 ±0.01	130	**	0.20 ±0.007	117	*	0.20 ±0.007	117	**
**Co** (μg g^{-1} DW)	0.017 ±0.004	0.013 ±0.002	75		0.013 ±0.004	80		0.01 ±0.003	60	
**Cr** (μg g-1 DW)	2.1 ±0.2	1.64 ±0.1	77		1.71 ±0.1	80		1.33 ±0.02	63	
**Cu** (μg g^{-1} DW)	11.6 ±0.2	11.6 ±0.6	99		12.4 ±0.2	107		11.16 ±0.26	95	
**Fe** (μg g^{-1} DW)	106 ±1	109 ±1.3	102		110 ±3.5	104		103 ±2	97	
**K** (mg g^{-1} DW)	41.1 ±0.3	44.4 ±0.6	108	**	45.4 ±1.3	110	*	43.8 ±0.6	106	**
**Mg** (mg g^{-1} DW)	3.3 ±0.05	3.1 ±0.12	96		3.0 ±0.06	92		2.9 ±0.06	91	**
**Mn** (μg g^{-1} DW)	136 ±2.4	146 ±5	107		140 ±5.5	103		132 ±2.5	97	
**Mo** (μg g^{-1} DW)	0.52 ±0.004	0.56 ±0.007	109	**	0.55 ±0.01	107	*	0.60 ±0.01	116	**
**Na** (mg g^{-1} DW)	136 ±10	198 ±7	145	**	191 ±9	140	**	227 ±6	167	***
**Ni** (μg g^{-1} DW)	1.0 ±0.2	1.1 ±0.13	102		1.19 ±0.07	110		0.93 ±0.02	86	
**P** (mg g^{-1} DW)	5.2 ±0.07	5.8 ±0.06	111	***	5.84 ±0.02	111	**	5.74 ±0.07	109	**
**Pb** (μg g^{-1} DW)	0.28 ±0.02	0.22 ±0.01	77		0.25 ±0.02	87		0.20 ±0.02	72	
**S** (mg g^{-1} DW)	2.65 ±0.01	2.68 ±0.01	101		2.69 ±0.05	101		2.69 ±0.02	101	
**Zn** (μg g^{-1} DW)	43.0 ±0.4	50.4 ±0.8	117	***	49.4 ±0.5	115	***	48.2 ±0.6	112	***

Supplemental Table S2. Element concentration in seeds of transgenic maize plants. Element content was analyzed from seeds of soil-grown transgenic and wild-type plants. Four biological replicates were analyzed for each genotype. Each biological replicate contained seeds from 2-3 plants. The statistical significance of differences from wild type was calculated using two-tailed Student's *t*-test (*, $p < 0.05$; **, $p < 0.01$; *** $p < 0.001$). DW, dry weight; NTC, non-transgenic control. Part of the data are shown in Figure 6.

	**NTC**	***pRCC3:CKX1-A2***			***pRCC3:CKX1-B9***			***pRCC3:CKX1-C4***		
**Element**	Mean ±SE	Mean ±SE	% NTC	*t*-test	Mean ±SE	% NTC	*t*-test	Mean ±SE	% NTC	*t*-test
**Al** (µg g^{-1} DW)	4.7 ±1.8	2.9 ±1.6	62		14.8 ±5.2	315		8.7 ±3.3	186	
**B** (µg g^{-1} DW)	3.1 ±0.4	2.91 ±0.1	95		2.41 ±0.2	78		2.87 ±0.1	92	
**Ca** (µg g^{-1} DW)	189 ±46	195 ±75	103		90 ±6	48		87 ±9	46	
**Cd** (mg g^{-1} DW)	0.012 ±0.002	0.013 ±0.002	100		0.012 ±0.002	100		0.01 ±0.000	80	
**Co** (µg g^{-1} DW)	0.012 ±0.004	0.018 ±0.002	140		0.017 ±0.008	140		0.017 ±0.01	140	
**Cr** (µg g-1 DW)	1.29 ±0.82	0.27 ±0.03	21		0.33 ±0.03	26		0.28 ±0.02	22	
**Cu** (µg g^{-1} DW)	4.2 ±0.2	4.7 ±0.8	113		7.3 ±0.6	174	**	5.3 ±0.5	128	
**Fe** (µg g^{-1} DW)	41.5 ±4.8	41.4 ±2.4	100		45.5 ±7.3	110		49 ±5.6	118	
**K** (mg g^{-1} DW)	4.2 ±0.05	3.6 ±0.05	86	***	3.5 ±0.04	84	***	4.3 ±0.25	102	
**Mg** (mg g^{-1} DW)	1.26 ±0.04	1.29 ±0.02	102		1.25 ±0.02	99		1.36 ±0.01	108	
**Mn** (µg g^{-1} DW)	12.0 ±1.1	17.8 ±0.5	149	**	13.6 ±0.5	113		15.6 ±0.3	130	*
**Mo** (µg g^{-1} DW)	0.29 ±0.05	0.25 ±0.008	85		0.28 ±0.008	97		0.32 ±0.01	104	
**Na** (mg g^{-1} DW)	27.5 ±6.7	14.5 ±1.1	53		114 ±18	414	**	49 ±18	180	
**Ni** (µg g^{-1} DW)	0.56 ±0.13	0.15 ±0.01	27		0.18 ±0.04	32		0.18 ±0.03	33	
**P** (mg g^{-1} DW)	3.19 ±0.08	3.17 ±0.04	99		3.14 ±0.04	99		3.30 ±0.04	103	
**Pb** (µg g^{-1} DW)	0.15 ±0.02	0.19 ±0.07	128		0.26 ±0.05	174		0.26 ±0.08	172	
**S** (mg g^{-1} DW)	1.49 ±0.04	1.40 ±0.01	94		1.47 ±0.03	99		1.54 ±0.04	104	
**Zn** (µg g^{-1} DW)	29.89 ±1.7	30.69 ±0.8	99		26.86 ±0.6	87		35.6 ±0.4	119	*

Supplemental Table S3. Sequences of primers used in the study.

Gene	Forward primer (5′- 3′)	Reverse primer (5′- 3′)
AtCKX1	ACGACCCTCTAGCGATTCT	CGGCAGTATTGATGCGTA
Zm EF1α	TGGGCCTACTGGTCTTACTACTGA	ACATACCCACGCTTCAGATCCT
Zm β-TUB	CTACCTCACGGCATCTGCTATGT	GTCACACACACTCGACTTCACG
ZmRR1	ACTGCGTGTGGTGCTATGTT	CCCCCAAATGTTAGCTCCTTGT
ZmRR2	CGAGGACTTCCTCCTCAAGC	CCAGATCCTCCATCCTTGCG
OsRCc3 promoter	TTAGAAGCAGTACGATCTTATTTGGTGGAGTTG	TGCTACGTACCCGAGATCGATCGATCACAA
